# Cognitive-enhancing and antioxidant activities of the aqueous extract from *Markhamia tomentosa* (Benth.) K. Schum. stem bark in a rat model of scopolamine

**DOI:** 10.1186/s12993-017-0123-6

**Published:** 2017-03-28

**Authors:** Radu Ionita, Paula Alexandra Postu, Galba Jean Beppe, Marius Mihasan, Brindusa Alina Petre, Monica Hancianu, Oana Cioanca, Lucian Hritcu

**Affiliations:** 1 Department of Biology, Alexandru Ioan Cuza University of Iasi, Bd. Carol I, No. 11, 700506 Iasi, Romania; 2 Laboratory of Animal Physiology, Faculty of Science, University of Yaoundé I, PO Box, 812, Yaoundé, Cameroon; 3 Department of Biological Sciences, Faculty of Science, University of Maroua, PO Box, 814, Maroua, Cameroon; 4 Department of Chemistry, Alexandru Ioan Cuza University of Iasi, Bd. Carol I, No. 11, 700506 Iasi, Romania; 5 Faculty of Pharmacy, University of Medicine and Pharmacy “Gr. T. Popa”, 16 University Str., 700115 Iasi, Romania

**Keywords:** *Markhamia tomentosa* stem bark extract, Scopolamine, Spatial memory, Oxidative stress, Acetylcholinesterase, Alzheimer’s disease

## Abstract

**Background:**

Plants of the genus *Markhamia* have been traditionally used by different tribes in various parts of West African countries, including Cameroun. *Markhamia tomentosa* (Benth.) K. Schum. (Bignoniaceae) is used as an antimalarial, anti-inflammatory, analgesic, antioxidant and anti-Alzheimer agent. The current study was undertaken in order to investigate its anti-amnesic and antioxidant potential on scopolamine-induced cognitive impairment and to determine its possible mechanism of action.

**Methods:**

Rats were pretreated with the aqueous extract (50 and 200 mg/kg, p.o.), for 10 days, and received a single injection of scopolamine (0.7 mg/kg, i.p.) before training in Y-maze and radial arm-maze tests. The biochemical parameters in the rat hippocampus were also assessed to explore oxidative status. Statistical analyses were performed using two-way ANOVA followed by Tukey’s post hoc test. *F* values for which p < 0.05 were regarded as statistically significant.

**Results:**

In the scopolamine-treated rats, the aqueous extract improved memory in behavioral tests and decreased the oxidative stress in the rat hippocampus. Also, the aqueous extract exhibited anti-acetylcholinesterase activity.

**Conclusions:**

These results suggest that the aqueous extract ameliorates scopolamine-induced spatial memory impairment by attenuation of the oxidative stress in the rat hippocampus.

## Background

Alzheimer’s disease (AD) is considered to be the most common form of dementia relating to memory and cognitive decline. AD is a progressive neurodegenerative disorder in which dementia symptoms gradually worsen over a number of years [1].

The biochemical hallmarks of AD include the accumulation of the amyloid-beta (Aβ) peptide oligomers and soluble hyperphosphorylated tau proteins [2]. AD is also accompanied by the loss of the cholinergic markers in vulnerable neurons and the degeneration of basal forebrain cortical cholinergic neurons in end–stage AD patients [3]. The memory loss and cognitive impairments are strongly related to changes in the acetylcholinesterase (AChE) activity [4]. Moreover, AChE can increase the rate of fibrillation by binding amyloid-β-associated proteins as potent amyloid-promoting factors [5]. Thus, the cholinergic hypothesis led to the development of clinically effective therapeutics for AD [6].

Scopolamine, a muscarinic acetylcholine receptor (MAChR) antagonist, can block the cholinergic function of the central nervous system by targeting M1AChR and M2AChR. It has been reported that scopolamine can induce anterograde memory impairment, particularly short-term memory and learning acquisition [7]. Moreover, scopolamine can significantly increase the activity of AChE and malondialdehyde (MDA) levels in the cortex and hippocampus, and oxidative stress in the brain [8, 9].

Oxidative stress is an important factor in the pathophysiology of neurodegenerative disorders, including AD [10]. Oxidative damage triggers the pathogenesis and cognitive disturbances in AD [11]. AD is highly related to cholinergic deficits and intracellular oxidative stress. Scopolamine-induced AD model is a valuable animal model for screening anti-AD drugs [12].

*Markhamia tomentosa* (Benth.) K. Schum. commonly known in west Cameroon as “bobedu, abbe or mawelu” is a shrub or tree that belongs to the family Bignoniaceae [13]. Previously, we demonstrated that the methanolic extract of *M. tomentosa* leaves (50, 100 and 200 mg/kg) possess in vivo analgesic and anti-inflammatory effects in healthy rats and mice as claimed by the traditional practitioners [13]. In addition, we suggested that anti-inflammatory and analgesic effects of the methanolic extract are attributed to the inhibition of serotonin, histamine, prostaglandin and morphinomimetic action.

The root bark of *M. tomentosa* has been in vitro screened for AChE and butyrylcholinesterase inhibitory activity [14, 15]. The authors suggested that this plant could be considered for further studies in the management of early stages of AD. Moreover, ethanolic extract of *M. tomentosa* leaves (50, 100 and 150 mg/kg) was reported to prevent gastric mucosal ulceration in stomachs of the Wistar rats supported a scientific base for the traditional use of this plant [16]. Ibrahim et al. [17] demonstrated that the leaf extract of *M. tomentosa* has shown antiproliferative and apoptosis profile on the HeLa cells, but not in the MCF-7 breast cancer cell line and normal Vero cell line. Furthermore, the methanolic extract of *M. tomentosa* possesses in vitro high antioxidant activity as evidenced by DPPH TLC screening [18]. In this study, the results of DPPH assay at 33.33 µg/mL indicated maximum antioxidant activity at 80%. The aforementioned results indicated that the extract of *M. tomentosa* could be a source of natural antioxidants useful for preventing oxidative stress damage with relevance AD condition.

Recently, Ibrahim et al. [14] reported in a review that the main phytochemical identified constituents of *Markhamia* species were phenylpropanoid glycosides, terpenoids, phytosterols, lignans, and flavonoids. Verbascoside, one of the main active phenylpropanoid glycoside from *M. tomentosa* leaves, was reported to reverse memory impairment induced by a combination of D-gal and AlCl_3_ in a senescent mouse model [19]. Isoverbascoside, another phenylpropanoid glycoside from *M. tomentosa* leaves, ameliorated cognitive deficits in AD-like rat model induced by administration of Aβ1–42 through blocking of amyloid deposition, reversing cholinergic and hippocampal dopaminergic neuronal function [20]. Oleanolic acid, a triterpene identified in the stem bark of *M. tomentosa*, exhibited neuroprotective effects and improved Aβ-induced memory deficits in mice [21, 22]. Palmitone, a lignin isolated from the stem heartwood of *M. tomentosa*, exhibited neuroprotective effects and prevents pentylenetetrazole-induced neuronal damage in the CA3 hippocampal region of the prepuberal Wistar rats [23]. Lapachol and its furano derivatives, a quinone isolated from the stem bark of *M. tomentosa*, displayed significant anxiolytic and antidepressant effects in mice [24]. Finally, luteolin, a flavonoid identified from the *M. tomentosa* leaves, ameliorated memory impairment in streptozotocin-induced AD rat model [25]. Although the identified compounds from *M. tomentosa* had significant activity in AD, there is no study clarifying the possible cognitive-enhancing and antioxidant potentials of the aqueous extract from *M. tomentosa* stem bark in a rat model of scopolamine. Therefore, we investigated the possible memory-enhancing effects of the aqueous extract from *M. tomentosa* stem bark in memory-impaired rats and its possible mechanism on the levels of biochemical parameters in the rat hippocampus of the scopolamine model.

## Methods

### Plant collection and extraction

*Markhamia tomentosa* (Benth.) K. Schum. (Bignoniaceae) stem bark was collected from Yaoundé, Cameroon in June 2010. Identification and authentication of the plant material were done at the National Herbarium, Yaoundé, Cameroon where a voucher specimen (Nº1974/SRFK) was registered and deposited for ready reference. Air-dried stem bark of *M. tomentosa* was reduced to fine powder (1000 g) and macerated in 10 L of distilled water for 48 h at room temperature and then the mixture was filtered through Whatman filter paper no. 3. The aqueous extract was then lyophilized to obtain powder used for our various tests. Percentage yield (w/w) of 3.70% was obtained. The dried extract was dissolved in distilled water and administered by gastric gavage to animals at the doses of 50 and 200 mg/kg body weight.

### HPLC–DAD analysis

HPLC analysis of the aqueous extract from *M. tomentosa* stem bark was performed using a Thermo UltiMate3000 gradient chromatograph equipped with quaternary pumps controlled by Chromeleon interface, an autosampler and multidiode array detector (DAD). Solvents were filtered using a Millipore system and analysis was performed on an Accucore XL C18 column (150 × 4.6 mm, 4 µm). All the samples were filtered through 0.22 µm filter before being analyzed. The mobile phase was acetonitrile (A) and water containing 0.1% acetic acid (B) and the composition gradient was: 10–23% (A) in 5 min; 23% (A) isocratic for 10 min and then 23–35% (A) in 12 min; 35–70% (A) for 5 min. The injection volume was 20 µL scanning absorbance wavelengths from 240 to 520 nm, typical for phenols including flavonols, flavones, hydroxycinnamic acids, and anthocyanins. The flow rate increased from 0.2 to 1 mL/min. HPLC grade solvents and bidistilled water were used in the chromatographic studies. All chromatographic experiments were performed at 25 °C. Standard curves for authentic samples of the polyphenols were obtained from purchased reagents (Sigma Chemical Co., USA) of analytical or high-performance liquid chromatography (HPLC) grade. Each solution was injected in triplicate and the calibration curves were constructed with the averages. A stock solution of the investigated samples was obtained by dissolving 1.915 mg of dry extract in 1 mL of HPLC grade methanol. Different amounts (1–20 µL) were injected by the autosampler. The final results represent the mean of three to five measurements.

### Animals

Twenty male Wistar rats weighing 350 ± 10 g (4–5-month-old) at the start of the experiment were used. The animals were housed in a temperature and light-controlled room (22 °C, a 12-h cycle starting at 08:00 h) and were fed and allowed to drink water ad libitum. The experiments were conducted in the quiet laboratory between hours of 10:00–16:00 h. The rats were divided into four groups (five animals per group): (1) the control group received the distilled water treatment; (2) the scopolamine (Sco)-alone-treated group received the distilled water treatment, as negative control; (3) the scopolamine-treated group received 50 mg/kg of the aqueous extract from *M. tomentosa* stem bark treatment [Sco+ME (50 mg/kg)]; (4) the scopolamine-treated group received 200 mg/kg of the aqueous extract from *M. tomentosa* stem bark treatment [Sco+ME (200 mg/kg)]. The aqueous extract from *M. tomentosa* stem bark was dissolved in distilled water. The administration of the distilled water and the aqueous extract was performed by 15-gauge oral gavage needle (Instech, Plymouth Meeting, PA). The volume administered was 10 mL/kg of body weight, daily, for 10 consecutive days. Moreover, animals received extract treatment during training in the Y-maze and the radial arm-maze tasks. The aqueous extract doses (50 and 200 mg/kg) used in this experiment were chosen since they have been demonstrated by our group to provide significant analgesic and anti-inflammatory effects as claimed by traditional healers [13]. Scopolamine hydrobromide (Sigma-Aldrich, Germany) was dissolved in an isotonic solution (0.9% NaCl) and 0.7 mg/kg scopolamine was injected intraperitoneally (i.p.), 30 min before the behavioral testing in the Y-maze and radial arm-maze tasks. Rats were treated in accordance with the guidelines of animal bioethics from the act on animal experimentation and animal health and welfare from Romania and all procedures were in compliance with Directive 2010/63/EU of the European Parliament and of the Council of 22 September 2010 on the protection of animals used for scientific purposes.

### Y-maze task

Short-term memory was assessed by spontaneous alternation behavior in the Y-maze task. The Y-maze used in the present study consisted of three arms (35 cm long, 25 cm high and 10 cm wide) and an equilateral triangular central area. 30 min after the aqueous extract from *M. tomentosa* stem bark administration, rats were placed at the end of one arm and allowed to move freely through the maze for 8 min. An arm entry was counted when the hind paws of the rat were completely within the arm. Spontaneous alternation behavior was defined as entry into all three arms on consecutive choices. The number of maximum spontaneous alternation behaviors was then the total number of arms entered minus two and percent spontaneous alternation was calculated as (actual alternations/maximum alternations) × 100. The maze was cleaned with a 10% ethanol solution and dried with a cloth before the next animal was tested. Spontaneous alternation behavior is considered to reflect spatial working memory, which is a form of short-term memory [26, 27].

### Radial arm-maze task

The radial arm-maze used in the present study consisted of eight arms, numbered from 1 to 8 (48 × 12 cm), extending radially from a central area (32 cm in diameter). The apparatus was placed 40 cm above the floor and surrounded by various extra-maze visual cues placed at the same position during the study. At the end of each arm, there was a food cup that had a single 50 mg food pellet. Prior to the performance of the maze task, the animals were kept on restricted diet and body weight was maintained at 85% of their free-feeding weight over a week period, with water being available ad libitum. Before the actual training began, three or four rats were simultaneously placed in the radial maze and allowed to explore for 5 min and take food freely. The food was initially available throughout the maze but was gradually restricted to the food cup. The animals were trained for 4 days to run to the end of the arms and consume the bait. To evaluate the basal activity of rats in radial eight arm-maze, the rats were given one training trial per day to run to the end of the arms and consume the bait. The training trial continued until all the five baits had been consumed or until 5 min has elapsed. After adaptation, all rats were trained with one trial per day. Briefly, 30 min after the aqueous extract from *M. tomentosa* stem bark administration, each animal was placed individually in the center of the maze and subjected to working and reference memory tasks, in which same 5 arms (no. 1, 2, 4, 5 and 7), were baited for each daily training trial. The other 3 arms (no. 3, 6 and 8) were never baited. An arm entry was counted when all four limbs of the rat were within an arm. Measures were made of the number of working memory errors (entering an arm containing food, but previously entered), reference memory errors (entering an arm that was not baited). The maze was cleaned with a 10% ethanol solution and dried with a cloth before the next animal was tested. Reference memory is regarded as a long-term memory for information that remains constant over repeated trials (memory for the positions of baited arms), whereas working memory is considered a short-time memory in which the information to be remembered changes in every trial (memory for the positions of arms that had already been visited in each trial) [26, 28].

### Biochemical parameter assay

After the behavioral tests, all rats were deeply anesthetized (using sodium pentobarbital, 100 mg/kg b.w., i.p., Sigma-Aldrich, Germany), decapitated and whole brains were removed. The hippocampi were carefully excised. Each of the hippocampal samples was weighed and homogenized (1:10) with Potter Homogenizer coupled with Cole-Parmer Servodyne Mixer in ice-cold 0.1 M potassium phosphate buffer (pH 7.4), 1.15% KCl. The homogenate was centrifuged (15 min at 960×*g*) and the supernatant was used for assays of AChE, SOD, and GPX specific activities, the total content of reduced GSH, protein carbonyl, and MDA levels.

### Determination of hippocampal AChE activity

The activity of acetylcholinesterase (AChE) in the rat hippocampus was determined according to the method of Ellman et al. [29] using acetylthiocholine (ATC) as artificial substrate [30, 31]. The reaction mixture (600 µL final volume) contained 0.26 M phosphate buffer with pH 7.4, 1 mM 5,5′-dithio-bis-2-nitrobenzoic acid (DTNB) and 5 mM ATC chloride. The assay was started by adding supernatant and following the developing of the yellow color at 412 nm for 10 min at room temperature. Suitable controls were performed for the non-enzymatic hydrolysis of ATC. The enzyme activity is expressed as nmol of ACT/min per/mg of protein.

### Determination of hippocampal SOD activity

The activity of superoxide dismutase (SOD, EC 1.15.1.1) was assayed by monitoring its ability to inhibit the photochemical reduction of nitroblue tetrazolium (NBT). Each 1.5 mL reaction mixture contained 100 mM TRIS/HCl (pH 7.8), 75 mM NBT, 2 μM riboflavin, 6 mM EDTA and 200 μL of supernatant. Monitoring the increase in absorbance at 560 nm followed the production of blue formazan. One unit of SOD is defined as the quantity required to inhibit the rate of NBT reduction by 50% as previously described by Winterbourn et al. [32, 33]. The enzyme activity is expressed as units/mg protein.

### Determination of hippocampal GPX activity

Glutathione peroxidase (GPX, E.C. 1.11.1.9) activity was analyzed by a spectrophotometric assay. A reaction mixture consisting of 1 mL of 0.4 M phosphate buffer (pH 7.0) containing 0.4 mM EDTA, 1 mL of 5 mM NaN_3_, 1 mL of 4 mM glutathione (GSH), and 200 μL of supernatant was pre-incubated at 37 °C for 5 min. Then 1 mL of 4 mM H_2_O_2_ was added and incubated at 37 °C for further 5 min. The excess amount of GSH was quantified by the 5,5′-dithiobis-2-nitrobenzoic acid (DTNB) method as previously described by Sharma and Gupta [34, 35]. One unit of GPX is defined as the amount of enzyme required to oxidize 1 nmol GSH/min. The enzyme activity is expressed as units/mg protein.

### Total hippocampal content of reduced GSH

Glutathione (GSH) was measured following the method of Fukuzawa and Tokumura [36, 37]. 200 µL of supernatant was added to 1.1 mL of 0.25 M sodium phosphate buffer (pH 7.4) followed by the addition of 130 µL DTNB 0.04%. Finally, the mixture was brought to a final volume of 1.5 mL with distilled water and absorbance was read in a spectrophotometer at 412 nm and results were expressed as µg GSH/µg protein.

### Determination of hippocampal protein carbonyl level

The extent of protein oxidation in the hippocampus was assessed by measuring the content of protein carbonyl groups, using 2,4-dinitrophenylhydrazine (DNPH) derivatization as described by Oliver et al. [38] and following the indications of Luo and Wehr [39, 40]. Basically, the supernatant fraction was divided into two equal aliquots containing approximately 2 mg of protein each. Both aliquots were precipitated with 10% trichloroacetic acid (TCA, w/v, final concentration). One sample was treated with 2 N HCl, and the another sample was treated with an equal volume of 0.2% (w/v) DNPH in 2 N HCl. Both samples were incubated at 25 °C and stirred at 5 min intervals. The samples were then reprecipitated with 10% TCA (final concentration) and subsequently extracted with ethanol–ethyl acetate (1:1, v/v) and then reprecipitated at 10% TCA. The pellets were carefully drained and dissolved in 6 M guanidine hydrochloride with 20 mM sodium phosphate buffer, pH 6.5. Insoluble debris was removed by centrifugation at 13,000×*g* at 4 °C. The absorbance at 370 nm of the DNPH-treated sample vs. the HCl control was recorded, and the results are expressed as nmols of DNPH incorporated/mg of protein based on an average absorptivity of 21/mM cm for most aliphatic hydrazones.

### Determination of MDA level

Malondialdehyde (MDA), which is an indicator of lipid peroxidation, was spectrophotometrically measured by using the thiobarbituric acid assay as previously described by Ohkawa et al. [41, 42]. 200 μL of supernatant was added and briefly mixed with 1 mL of 50% trichloroacetic acid in 0.1 M HCl and 1 mL of 26 mM thiobarbituric acid. After vortex mixing, samples were maintained at 95 °C for 20 min. Afterward, samples were centrifuged at 960×*g* for 10 min and supernatants were read at 532 nm. A calibration curve was constructed using MDA as standard and the results were expressed as nmol/mg protein.

### Estimation of protein concentration

Estimation of protein was done using a bicinchoninic acid (BCA) protein assay kit (Sigma-Aldrich, Germany). The BCA protein assay is a detergent-compatible formulation based on BCA for the colorimetric detection and quantification of total protein, as previously described by Smith et al. [43, 44].

### Statistical analysis

Behavioral scores within Y-maze and radial arm-maze tasks and biochemical data were analyzed by two-way analysis of variance (ANOVA) followed by Tukey post hoc test using GraphPad Prism 6 software for Windows, La Jolla California USA. In order to evaluate differences between groups in the radial arm-maze task, separate repeated-measures ANOVA were calculated on the number of working memory errors and the number of reference memory errors with group [Control, Sco, Sco+ME (50 mg/kg) and Sco+ME (200 mg/kg)] as between-subject factor and days (1–7) as within-subjects factors. All results are expressed as a mean ± standard error of the mean (SEM). *F* values for which p < 0.05 were regarded as statistically significant. Pearson’s correlation coefficient and regression analysis were used in order to evaluate the connection between behavioral measures, the antioxidant defense, and lipid peroxidation.

## Results

### Phytochemical screening

A stock solution of the investigated samples was obtained by dissolving 1.915 mg of dry extract in 1 mL of HPLC grade methanol. Different amounts (1–20 µL) were injected by the autosampler. The final results represent the mean of 3–5 measurements.

**Fig. 1:**
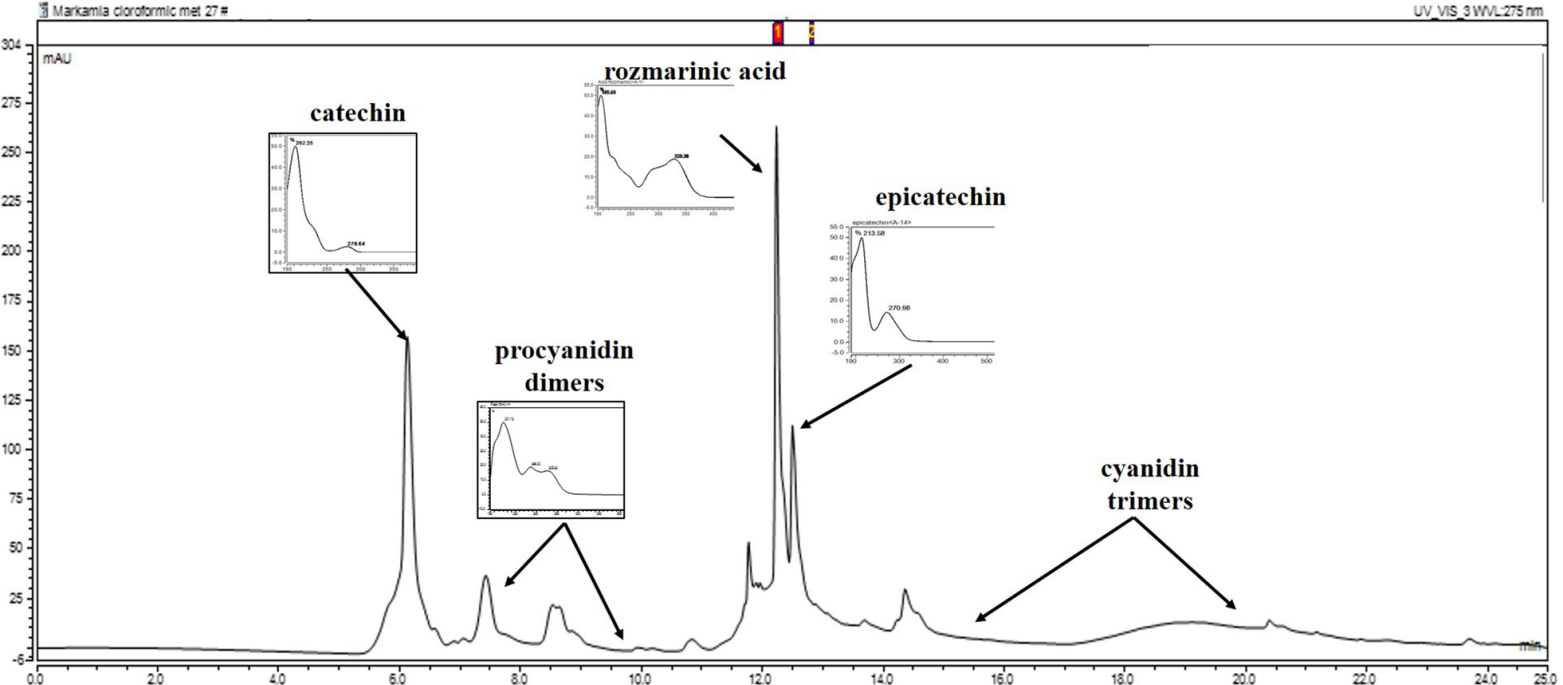
HPLC chromatogram the aqueous extract from *Markhamia tomentosa* stem bark. The major identified compounds were rozmarinic acid, (+)-catechin, procyanidin dimer, (−)-epicatechin and cyanidin trimmers

Regarding *M. tomentosa* stem bark there are no studies available in regards to the chemical composition of this vegetal product. Although some researchers have evaluated the biologic properties of *M. tomentosa* leaves extracts, there is no data confirming the presence of specific compounds, but rather major groups of substances. Thus, from the 14 standards used, we were able to identify and quantify catechin, epicatechin, rosmarinic acid and several catechin/epicatechin derivatives (without being able to specify which) as indicated in the chromatogram below (Fig. 1).

The amounts detected/mg of dry extract were: 541.5 µg/mg rozmarinic acid; 11.37 µg/mg (+)-catechin; 15.86 µg/mg procyanidin dimer; 42.47 µg/mg (−)-epicatechin and 14.65 µg/mg cyanidin trimmers. Such chemical composition is related to strong antioxidant and radical chelating activities, as many researchers state the importance of catechin derivatives in protective mechanisms [45].

### Effect of the aqueous extract from *Markhamia tomentosa* stem bark on behavioral performance

**Fig. 2:**
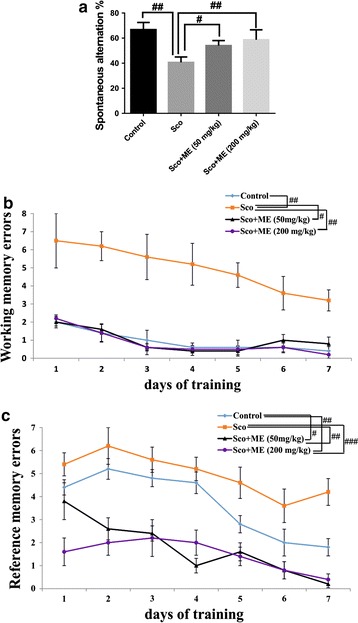
Effects of the aqueous extract from *Markhamia tomentosa* stem bark (50 and 200 mg/kg) in the Y-maze on spontaneous alternation  % (**a**) and on the working memory errors (**b**) and the reference memory errors (**c**) during 7 days training in radial arm-maze task in the scopolamine-treated rats. Values are mean ± SEM (n = 5 animals per group). For Tukey’s post hoc analysis—^##^Control vs. Sco: p < 0.001, ^#^Sco vs. Sco+ME (50 mg/kg): p < 0.01 and ^##^Sco vs. Sco+ME (200 mg/kg): p < 0.001 (**a**), ^##^Control vs. Sco: p < 0.0001, ^#^Sco vs. Sco+ME (50 mg/kg): p < 0.001 and ^##^Sco vs. Sco+ME (200 mg/kg): p < 0.0001 (**b**) and ^#^Control vs. Sco+ME (50 mg/kg): p < 0.001, ^##^Control vs. Sco+ME (50 mg/kg): p < 0.001, ^##^Sco vs. Sco+ME (50 mg/kg): p < 0.0001 and ^###^Sco vs. Sco+ME (200 mg/kg): p < 0.0001 (**c**)

In the Y-maze test, significant overall differences between groups (F(3, 16) = 3.68, p < 0.01) on the spontaneous alternation percentage were evidenced (Fig. 2a). The results suggest that scopolamine treatment decreased the spontaneous alternation percentage (p < 0.01) as compared to control group. The scopolamine treated rats with both doses of studied extract, but especially the dose of 200 mg/kg, displayed significant differences (p < 0.001) for spontaneous alternations percentage as compared to scopolamine-alone treated group.

In the radial arm-maze task, significant overall differences between groups (F(3,16) = 43.64, p < 0.0001) on the working memory were evidenced (Fig. 2b). Scopolamine significantly increased (p < 0.0001) the working memory errors as compared to control group. The scopolamine treated rats with both doses of the aqueous extract showed a decreased (p < 0.0001) working memory errors as compared to scopolamine-alone treated group. Moreover, repeated-measures ANOVA revealed a significant group difference (F(3,252) = 34.62, p < 0.0001) for the working memory errors. Additionally, Tukey’s post hoc analysis revealed a significant difference between group vs. working memory errors (p < 0.0001).

ANOVA revealed significant overall differences between groups (F(3,16) = 17.78, p < 0.0001) on the reference memory (Fig. 2c). Scopolamine significantly increased (p < 0.01) the reference memory errors as compared to control group. Rats in the scopolamine group pretreated with the extract showed a decreased (p < 0.0001) reference memory errors especially at the dose of 200 mg/kg as compared to scopolamine-alone treated group. Moreover, repeated-measures ANOVA revealed a significant group difference (F(3,252) = 3.31, p < 0.01) for the reference memory errors. Additionally, Tukey’s post hoc analysis revealed a significant difference between group vs. reference memory errors (p < 0.0001).

### Effect of the aqueous extract from *Markhamia tomentosa* stem bark on the AChE activity

**Fig. 3:**
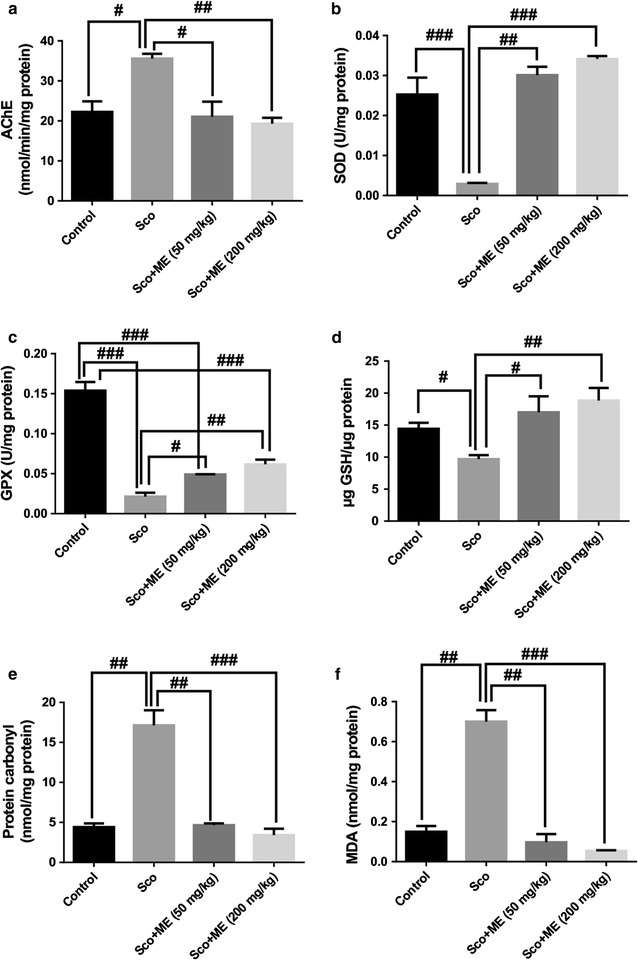
Effects of the aqueous extract from *Markhamia tomentosa* stem bark (50 and 200 mg/kg) on AChE (**a**), SOD (**b**) and GPX (**c**) specific activities, on reduced GSH (**d**), protein carbonyl (**e**) and MDA (**f**) levels in the scopolamine-treated rats. Values are mean ± SEM. (n = 5 animals per group). For Tukey’s post hoc analysis—^#^Control vs. Sco: p < 0.01, ^#^Sco vs. Sco+ME (50 mg/kg): p < 0.01 and ^##^Sco vs. Sco+ME (200 mg/kg: p < 0.001 (**a**), ^###^Control vs. Sco: p < 0.0001, ^##^Sco vs. Sco+ME (50 mg/kg): p < 0.001 and ^###^Sco vs. Sco+ME (200 mg/kg): p < 0.0001 (**b**), ^###^Control vs. Sco: p < 0.0001, ^###^Control vs. Sco+ME (50 mg/kg): p < 0.0001, ^###^Control vs. Sco+ME (200 mg/kg): p < 0.0001, ^#^Sco vs. Sco+ME (50 mg/kg): p < 0.01 and ^##^Sco vs. Sco+ME (200 mg/kg): p < 0.001 (**c**), ^#^Control vs. Sco: p < 0.01, ^#^Sco vs. Sco+ME (50 mg/kg): p < 0.01 and ^##^Sco vs. Sco+ME (200 mg/kg): p < 0.001 (**d**), ^##^Control vs. Sco: p < 0.0001, ^##^Sco vs. Sco+ME (50 mg/kg): p < 0.0001 and ^###^Sco vs. Sco+ME (200 mg/kg): p < 0.00001 (**e**) and ^##^Control vs. Sco: p < 0.0001, ^##^Sco vs. Sco+ME (50 mg/kg): p < 0.0001 and ^###^Sco vs. Sco+ME (200 mg/kg): p < 0.00001 (**f**)

For the AChE specific activity estimated in the rat hippocampal homogenates, significant overall differences between groups (F(3,16) = 9.00, p < 0.001) were evidenced (Fig. 3a). Scopolamine treatment increases the AChE specific activity (p < 0.01) as compared to control group, while administration of the studied extract, in a dose of 50 mg/kg (p < 0.01), but especially at the dose of 200 mg/kg, decreased the AChE specific activity (p < 0.001) as compared to the scopolamine-alone treated group.

### Effect of the aqueous extract from *Markhamia tomentosa* stem bark on the SOD and GPX activities

For the SOD specific activity estimated in the rat hippocampal homogenates, significant overall differences between groups (F(3,16) = 34.41, p < 0.0001) were noticed (Fig. 3b). While scopolamine treatment decreased SOD specific activity (p < 0.0001) as compared to control group, the administration of the aqueous extract in a dose of 50 mg/kg, significantly reverse the SOD activity (p < 0.001), but especially at the dose of 200 mg/kg (p < 0.0001), as compared to scopolamine-alone treated group.

In the rat hippocampal homogenates, significant overall differences between groups (F(3, 16) = 75.15, p < 0.0001) were evidenced for the GPX specific activity (Fig. 3c). Scopolamine group displayed markedly decline for the GPX specific activity (p < 0.0001) compared with control group. In addition, the results revealed that administration of the aqueous extract in a dose of 50 mg/kg (p < 0.01), but especially at the dose of 200 mg/kg (p < 0.001) could effectively reverse the GPX specific activity in scopolamine-induce decreasing of the GPX specific activity.

### Effect of the aqueous extract from *Markhamia tomentosa* stem bark on the total content of reduced GSH, protein carbonyl, and MDA levels

In the rat hippocampal homogenates, significant overall differences between groups (F(3, 16) = 57.92, p < 0.01) were displayed for the total content of reduced GSH (Fig. 3d). The total content of reduced GSH decreased in scopolamine group (p < 0.01) as compared to control group. Treatment with both doses of 50 mg/kg (p < 0.01) and 200 mg/kg (p < 0.001) of the aqueous extract to scopolamine administered rats significantly increased GSH content over normal levels.

For the protein carbonyl level measured in the rat hippocampal homogenates, significant overall differences between groups (F(3, 16) = 37.84, p < 0.0001) were revealed (Fig. 3e). Protein carbonyl level showed a significant increase (p < 0.0001) as compared to control group. Treatment of the aqueous extract, either with 50 mg/kg (p < 0.0001) and 200 mg/kg (p < 0.00001) to scopolamine administered rats significantly reduced protein carbonyl level close to normal levels.

For the lipid peroxidation (MDA) level measured in the rat hippocampal homogenates, significant overall differences between groups (F(3, 16) = 62.99, p < 0.0001) were evidenced (Fig. 3f). Administration of scopolamine resulted in increasing of the MDA level (p < 0.0001) as compared to control group. The results also revealed that in the scopolamine treated group, administration of the aqueous extract, at the doses of 50 mg/kg (p < 0.0001) and 200 mg/kg (p < 0.00001), markedly decreased MDA level under normal levels.

**Fig. 4:**
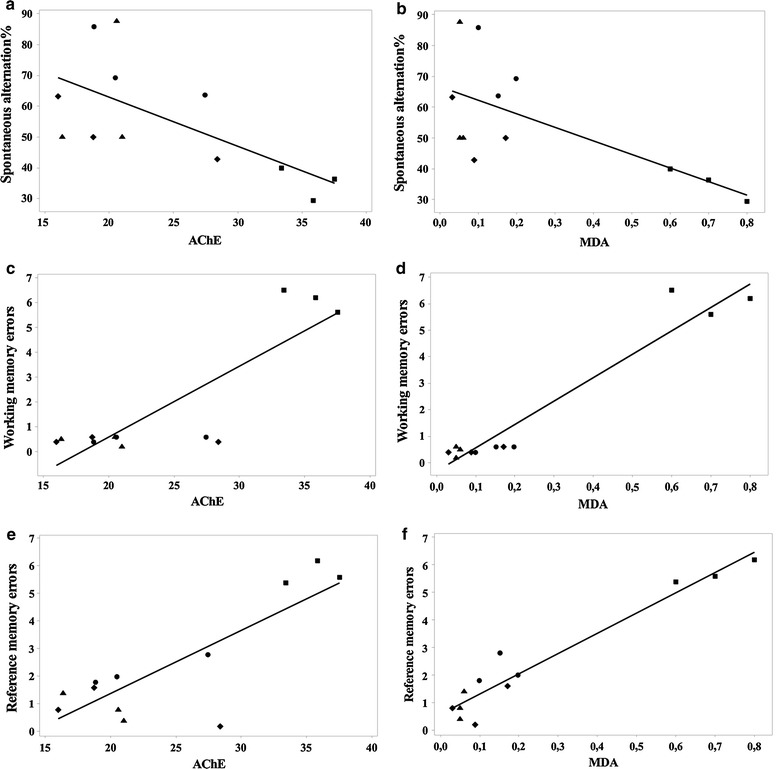
Pearson’s correlation between the spontaneous alternation percentage vs. AChE (**a**), spontaneous alternation percentage vs. MDA (**b**), working memory errors vs. AChE (**c**), working memory errors vs. MDA (**d**), reference memory errors vs. AChE (**e**) and reference memory errors vs. MDA (**f**) in control group (*filled circle*), scopolamine alone treated-group (*filled square*), Sco+ME (50 mg/kg) group (*filled diamond*) and Sco+ME (200 mg/kg) group (*filled triangle*)

Importantly, when linear regression was determined, significant correlations between the spontaneous alternation percentage vs. AChE (n = 20, r = −0.657, p < 0.01) (Fig. 4a), spontaneous alternation percentage vs. MDA (n = 20, r = −0.664, p < 0.01) (Fig. 4b), working memory errors vs. AChE (n = 20, r = 0.856, p < 0.0001) (Fig. 4c), working memory errors vs. MDA (n = 20, r = 0.969, p < 0.001) (Fig. 4d), reference memory errors vs. AChE (n = 20, r = 0.826, p < 0.001) (Fig. 4e) and reference memory errors vs. MDA (n = 20, r = 0.966, p < 0.0001) (Fig. 4f) were evidenced.

**Fig. 5:**
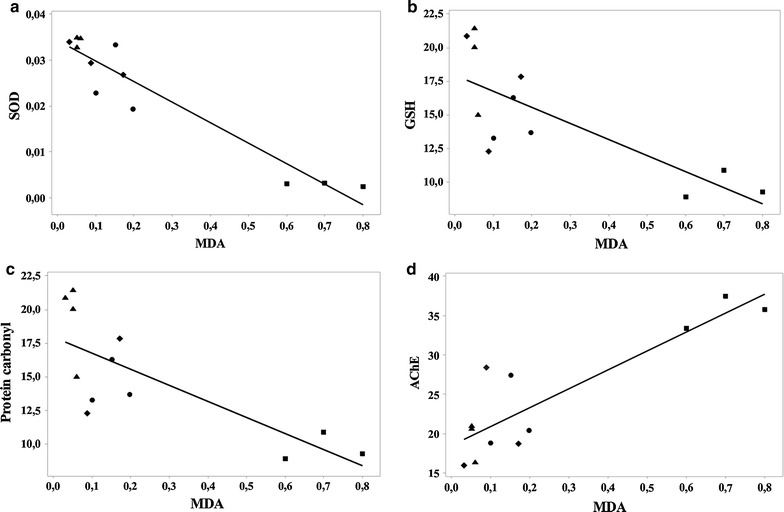
Pearson’s correlation between SOD vs. MDA (**a**), GSH vs. MDA (**b**), protein carbonyl vs. MDA (**c**) and AChE vs. MDA (**d**) in control group (*filled circle*), scopolamine alone treated-group (*filled square*), Sco+ME (50 mg/kg) group (*filled diamond*) and Sco+ME (200 mg/kg) group (*filled triangle*)

Additionally, a significant correlation was evidenced by determination of the linear regression between SOD vs. MDA (n = 20, r = −0.953, p < 0.0001) (Fig. 5a), GSH vs. MDA (n = 20, r = −0.766, p < 0.001) (Fig. 5b), protein carbonyl vs. MDA (n = 20, r = 0.877, p < 0.001) (Fig. 5c) and AChE vs. MDA (n = 20, r = 0.877, p < 0.0001) (Fig. 5d). However, the significant correlation between MDA levels and behavioral measures, as well as MDA and biochemical measures, consistently displayed three rats all scopolamine-treated that were driving the significant relationship.

## Discussion

In the present study, a series of experiments were designed in order to investigate the cognitive improvement of the aqueous extract from *M. tomentosa* stem bark in a scopolamine-induced a rat model of cognitive impairment in vivo.

Scopolamine is a muscarinic acetylcholine receptor (MAChR) antagonist known to block signals underlying memory [46]. Our results are in line with previous data showing that the rats with a model of scopolamine-impaired memory significantly decreased their scores during training sessions within Y-maze and radial arm-maze tests [47–49]. Administration of the aqueous extract from *M. tomentosa* stem bark at both doses improved the impairment effect of scopolamine on memory formation, suggesting that the aqueous extract could act as an unspecific enhancer of the cholinergic activity.

The possible underlying mechanism of the aqueous extract action could be the increase of the brain cholinergic receptor sensitivity or the decrease of the AChE activity. Sugisaki et al. [50] reported that hippocampal-dependent memory is dependent by the increasing of extracellular acetylcholine (ACh) level. Also, the cholinergic synaptic transmission could by impaired by an overexpression of AChE activity-induced decreasing of ACh level [51]. AChE activity estimated in the rat hippocampal homogenates was significantly increased by scopolamine as compared to control group. The aqueous extract administration significantly decreased the AChE activity in the scopolamine-treated rats, suggesting that the aqueous extract may confer anti-amnesic effects. Similarly, strong inhibition of the brain AChE activity was evidenced by administration of different herbal extracts in the scopolamine treated-rats [52, 53].

As an argument supporting this mechanism, the HPLC–DAD analysis of the aqueous extract from *M. tomentosa* stem bark showed that the most important group of components isolated were water-soluble polyphenolic derivatives (catechins, hydroxycinnamic acid compounds), mainly rozmarinic acid (541.5 µg/mg of dry extract), (+)-catechin (11.37 µg/mg of dry extract), procyanidin dimer (15.86 µg/mg of dry extract), (−)-epicatechin (42.47 µg/mg of dry extract) and cyaniding trimmers (14.65 µg/mg of dry extract). We can thus suggest that the effect of the aqueous extract on memory formation may be due to the presence of polyphenolic compounds such as rosmarinic acid and (−)-epicatechin.

It has been reported that rosmarinic acid exerted various beneficial biological effects such as antioxidant and neuroprotective effects and anti-AChE activity [54–56]. Also, rosmarinic acid has positive effects on learning and memory in the SAMP8 mouse model of accelerated aging [57] and decreased memory deficits in ischemic mice [58]. Furthermore, Zhang et al. [59] demonstrated that epicatechin plus treadmill exercise are neuroprotective against moderate-stage amyloid precursor protein/presenilin 1 mice. Also, epicatechin display a potent anti-AChE activity as previously reported [60]. Tseng et al. [61] reported that (−)-epigallocatechin-3-gallate prevents the reserpine-induced impairment of short-term social memory in rats most probably through its powerful antioxidant activities.

One of the important mechanism in the development and progression of AD is oxidative stress. In the present study, scopolamine decreased SOD, GPX, and GSH and increased the MDA and protein carbonyl levels in the rat hippocampal homogenates. It has been documented that scopolamine administration induced a neurochemical alteration in the brain along with changes in oxidative status of the brain [9]. Thus, scopolamine created an imbalance between antioxidant and oxidant defense systems which may be responsible for observed impairment of memory in rats. Furthermore, many studies have reported that the scopolamine-induced amnesic rats show similar patterns of memory impairments and oxidative damage with amnestic mild cognitive impairment (MCI) patients [62]. Evidence suggested that different plant extracts have potent anti-amnesic effects that may be mediated by improving the brain oxidative status [63–65]. Consequently, the aqueous extract treatment restored the antioxidants status as evidenced by an increase of SOD, GPX, and GSH while the levels of MDA (lipid peroxidation) and protein carbonyl significantly decrease which supports its antioxidant property.

Moreover, we found a significant correlation between the spontaneous alternation percentage vs. AChE, spontaneous alternation percentage vs. MDA, working memory errors vs. AChE, working memory errors vs. MDA, reference memory errors vs. AChE, reference memory errors vs. MDA, SOD vs. MDA, GSH vs. MDA, protein carbonyl vs. MDA and AChE vs. MDA when linear regression was determined. These results could suggest that the increase of behavioral scores in the Y-maze and the radial arm-maze tests along with the decrease of AChE activity, and also the MDA content and protein carbonyl level could be correlated with the involvement of the aqueous extract in neuroprotection against scopolamine-induced oxidative stress generation in the rat hippocampus.

## Conclusions

In summary, the obtained results suggest that the aqueous extract from *M. tomentosa* stem bark (50 and 200 mg/kg) exerts its anti-amnesic effects through modulation of the antioxidant activity in the hippocampus of the rat model of scopolamine. Therefore, the aqueous extract may possibly be used as a promising natural product for the prevention of memory disorders and AD.

## Abbreviations

AD: Alzheimer’s disease
Aβ: amyloid-beta peptide
Aβ1–42: amyloid-beta peptide 1–42
ACh: acetylcholine
AChE: acetylcholinesterase
ANOVA: analysis of variance
BCA: bicinchoninic acid
DAD: multidiode array detector
DPPH TLC: 2,2-diphenyl-1-picrylhydrazyl thin-layer chromatography
DNPH: 2,4-dinitrophenylhydrazine
DTNB: 5,5′-dithiobis-2-nitrobenzoic acid
GPX: glutathione peroxidase
GSH: glutathione
HPLC: high-performance liquid chromatography
MAChR: muscarinic acetylcholine receptor
MDA: malondialdehyde
NBT: nitroblue tetrazolium
SOD: superoxide dismutase
